# Is dental autotransplantation underestimated and underused by Syrian dentists?

**DOI:** 10.3352/jeehp.2021.18.18

**Published:** 2021-08-04

**Authors:** Nuraldeen Maher Al-Khanati, Zafin Kara Beit

**Affiliations:** Department of Oral and Maxillofacial Surgery, Faculty of Dental Medicine, Damascus University, Damascus, Syria; Hallym University, Korea

**Keywords:** Autologous transplantation, Dentists, Tissue donors, Surveys and questionnaires, Syria

## Abstract

Dental autotransplantation (DAT) is a surgical procedure in which a donor's tooth is extracted and transplanted from one site to another in the same person. This treatment modality has received considerable attention worldwide in recent years due to its potential advantages over implants. A survey-based study evaluated dentists’ attitudes towards and practice of DAT in Damascus, Syria from September to December 2020. We asked respondents whether they considered this treatment modality when developing treatment plans and whether they view it viable. Only 73 of the 258 respondents (28.3%) stated that they considered DAT as a treatment option. Additionally, 153 respondents (59.3%) either did not view DAT as a viable treatment option or did not know whether it is viable. DAT was underestimated and underused among Syrian dentists. Given this gap in real-world knowledge and practice, academic dental institutions in Syria should place a greater focus on emerging evidence-based knowledge and protocols regarding this treatment option.

## Background/rationale

Dental autotransplantation (DAT) is a surgical procedure in which a donor's tooth is extracted and transplanted from one site to another in the same person [1]. In essence, it is a relatively old dental procedure that replaces a lost tooth with a natural one, rather than a prosthesis [1]. Although the concept of this treatment is not inherently novel, it has received considerable attention from researchers worldwide in recent years [2,3]. This raises the questions of whether clinicians are following recent research in the field, and whether studies on DAT have affected real-world clinical practice. As pointed out in previous studies, the technical difficulty of this treatment may be a barrier hindering its use by many dentists and oral surgeons, although emerging evidence is increasingly proving its viability [2,3].

## Objectives

This study presents the findings of a survey designed to evaluate dentists’ attitudes toward and practice of DAT in Damascus, Syria.

## Ethics statement

Ethical approval for this study was obtained from the Human Research Ethics Committee at Damascus University, Syria (no., 2019-1001). Study participants provided consent before participating in the survey. The questionnaire was preceded by a brief introduction explaining the purpose of the study and assuring participants that participation was voluntary.

## Study design

It is a cross-sectional descriptive study based on the survey.

## Setting

The questionnaire survey that collected information about attitudes towards and practice of DAT from Syrian dentists was done from September to December 2020.

## Participants

In total, 258 dentists (80.1%) out of total 322 who were practicing in Damascus completed a concise questionnaire. There were no exclusion criteria. The average (±standard deviation) age of the respondents was 33.9±10.4 years, and the mean duration of their professional experience practicing dentistry was 10.2±9.8 years. Among the participants, 63.6% (n=164) were men, 52.3% (n=135) worked in the private sector, and most of them (n=207, 80.2%) were dental specialists with a higher degree. Specialties included oral and maxillofacial surgery (n=85, 32.9%) and periodontology (n=33, 12.8%). Other dental specialties (e.g., prosthodontics, pedodontics, orthodontics and esthetic dentistry) accounted for 34.5% (n=89) of the participants. The remaining participants (n=51, 19.8%) were general dental practitioners (Table 1).

## Outcome variables

*Practice of DAT*: Respondents were asked whether they considered DAT in their treatment plans (yes/no), as well as how many cases there were in which they had considered DAT.

*Attitude towards DAT*: Respondents were asked why they did (or did not) consider DAT in their treatment plans (closed-ended question with pre-provided response possibilities), and whether they believed that DAT is a viable treatment option (yes/no). Respondents were asked to choose the first and most important reason, if more than one existed.

## Data source/measurement

Data were collected using hard-copy questionnaires gathered from dentists working in Damascus in both the private and public sectors. The questionnaire consisting of 2 yes/no binary items asking whether the respondent usually considers DAT when developing a treatment plan and whether he/she believes that DAT is a viable treatment option. Two other items were included to solicit information on the reasons for the respondents’ choices regarding the previously mentioned questions (Supplement 1).

## Statistical methods

Statistical tests were performed using IBM SPSS ver. 19.0 (IBM Corp., Armonk, NY, USA). A descriptive analysis, including frequency and mean±standard deviation of the study variables, was conducted. As the main survey questions had nominal, the chi-square test was used. Statistical significance was set at P<0.05.

## Main results

**Fig. 1.**
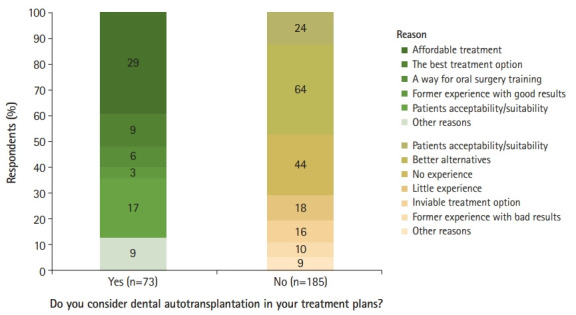
Respondents’ reasons for whether they considered dental autotransplantation while developing treatment plans to replace missing teeth.

Although many of the participants were oral and maxillofacial surgeons and periodontists, most of the respondents (n=185, 71.7%) did not consider DAT at all while developing treatment plans (χ^2^=48.62, P<0.001). The 80 respondents (43.2%) out of 185 dentists stated that there were much better alternatives to DAT and/or indicated that they considered this treatment modality to be comparatively inviable, although only 10 dentists reported previous experiences of negative outcomes using DAT as a reason for their negative answers (Fig. 1). In contrast, 62 respondents stated that they did not consider DAT in treatment planning because they had little or no experience with it, even though only 9 believed DAT to be the best treatment option (Fig. 1, Table 1, Dataset 1).

Out of the 73 respondents who answered that they did consider DAT in their treatment plans, 29 dentists stated that the main reason for this was the affordability of DAT compared to alternatives such as dental implants. Only 3 dentists reported that they usually considered DAT due to personal experiences of achieving good results with this treatment modality (Fig. 1). The study outcome variables were not significantly associated with respondents’ specialty, work sector, and gender (P>0.05), except for the question asking whether respondents believed that DAT is a viable treatment option, to which significantly more men than women responded “no” (P<0.001) (Table 1).

### Interpretation

This study aimed to shed light on dentists’ attitudes towards and practice of DAT in a Syrian sample. The key result of the present study is that most respondents (71.7%) did not consider DAT while developing treatment plans, and 59.3% of them either did not believe DAT that is a viable treatment option or did not know whether it is viable (Table 1).

A recent study concluded that DAT is a valid and esthetically satisfactory treatment in the maxilla, with high survival and success rates [2]. Even if the recipient site contains a chronic periapical lesion, immediate DAT can still be a proper treatment option as long as the recipient transplant bed is appropriately managed prior to DAT [3,4]. Moreover, a successful autotransplanted tooth with a functioning periodontium provides significant advantages over osseointegrated implants from a functional standpoint, such as proprioception, shock absorption, thermal feedback, and possible orthodontic movements [5].

This cross-sectional study revealed that Syrian clinicians frequently did not consider DAT as a treatment option due to a lack of experience and the perception that DAT is not viable. It seems that this issue is not limited to dentists in Syria. Internationally, authors believe that this treatment modality is underused and does not receive an appropriate amount of respect [6,7]. In India, oral and maxillofacial surgery residents were found to have a poor understanding of DAT and many misconceptions [7]. It has been reported that DAT is not a part of the curriculum at many dental teaching institutions and is not routinely practiced at many hospitals [6,7].

### Limitations and generalizability

The findings of a study must be viewed in light of its limitations. First, only dentists working in Damascus were included in this study; thus, our results may not necessarily be generalizable to dentists working in other Syrian cities or abroad. Nevertheless, similarities with prior studies’ findings may confer some validity beyond this study’s geographic scope. Moreover, this is a cross-sectional study that only provides preliminary evidence; further advanced research would be necessary to build upon these findings.

### Conclusion

Misconceptions about DAT exist, leading many dentists to refrain from practicing this treatment. Many dentists need to receive updated information to change the viewpoints that lead them to underestimate the outcomes of this treatment modality or exaggerate its challenges and complications. Emerging evidence-based knowledge and protocols regarding this treatment option should receive a greater focus in the curriculum at academic dental institutions, especially as it can be the most cost-effective option in many cases. Further cohort studies are warranted to assess quality improvements in dental educational curricula and their impact on dentists’ attitudes toward and practice of DAT after graduation.

## Figures and Tables

**Table 1.** General characteristics and answers of survey respondents

Characteristic	Do you consider DAT in your treatment plans?			Do you believe that DAT is a viable treatment option?				Total (%)
	Yes	No	P-value	Yes	No	I don’t know	P-value	
Gender			0.262				0.001	
Male	42	122		63	61	40		164 (63.6)
Female	31	63		42	12	40		94 (36.4)
Work sector			0.306				0.313	
Private	34	101		54	34	47		135 (52.3)
Public	39	84		51	39	33		123 (47.7)
Specialty			0.346				0.117	
GDP	10	41		25	14	12		51 (19.8)
OMFS	23	62		32	32	21		85 (32.9)
Perio	10	23		14	8	11		33 (12.8)
Other	30	59		34	19	36		89 (34.5)
Total (%)	73 (28.3)	185 (71.7)		105 (40.7)	73 (28.3)	80 (31.0)		258 (100.0)
P-value			0.001^a)^				0.037^b)^	
Chi-square			48.62				6.58	

DAT, dental autotransplantation, GDP, general dental practitioners, OMFS, oral and maxillofacial surgeons, Perio, periodontists.

a) The result of the one-way chi-square test of the categorical variable “Do you consider DAT in your treatment plans?” (expected values: “all categories equal”).

b) The result of the one-way chi-square test of the categorical variable “Do you believe DAT is a viable treatment option?” (expected values: “all categories equal”).

## Footnotes

**Data availability**
Data files are available from Harvard Dataverse: https://doi.org/10.7910/DVN/2VAOD9 Dataset 1. Response data of the survey to evaluate the attitudes towards and practice of dental autotransplantation by 258 dentists in Damascus, Syria.

## Supplementary materials

Data files are available from Harvard Dataverse: https://doi.org/10.7910/DVN/2VAOD9

Supplement 1. Questionnaire to dentists in Damascus, Syria on the dental autotransplantation in Arabic.

Supplement 2. Audio recording of the abstract.
