# Supplementary material for: Is dental autotransplantation underestimated and underused by Syrian dentists?
Source: J Educ Eval Health Prof. 2021 Aug 4;18:18. doi: 10.3352/jeehp.2021.18.18 (PMC8385419; doi:10.3352/jeehp.2021.18.18)
Supplement: Supplementary file 2 — Supplement 1. Questionnaire to dentists in Damascus, Syria on the dental autotransplantation in Arabic. [file jeehp-18-18-suppl1.pdf]

كلية طب الأســـنان
FACULTY OF DENTISTRY

استمارة استبيان مسحي لأطباء الأسنان في دمشق في سياق بحث علمي متعلق بالغرس الذاتي

الرمز: .... الاسم: .... الجنس: □ذكر □أنثى تاريخ الولادة:.............. الحالة الاجتماعية: □أعزب □متزوج

العمل:□خاص .... □قطاع عام .... الاختصاص: □ممارس عام □جراحة فكية □نسج حول سنية □غيرذلك.............

مكان الاختصاص: □جامعة دمشق □وزارة الصحة □وزارة الدفاع □غير ذلك...................... عدد سنوات ممارسة المهنة: ....

الرمز: ....

عادةً، هل تأخذ الزرع السني الذاتي Dental Autotransplantation بعين الاعتبار عند وضع خطط معالجة المرضى للتعويض عن الأسنان المفقودة؟ □ نعم □ لا

إذا كانت إجابتك السابقة "نعم" لطفاً أجب عن الأسئلة التالية

خلال السنوات الثلاثة الأخيرة، كم حالة قمت بتدبيرها باستخدام الزرع السني الذاتي؟ □0 □1 □2 □3 □4 □........

خلال السنوات الخمسة الأخيرة، كم حالة قمت بتدبيرها باستخدام الزرع السني الذاتي؟ □0 □1 □2 □3 □4 □........

خلال كل فترة ممارستك للمهنة، كم حالة قمت بتدبيرها باستخدام الزرع السني الذاتي؟ □0 □1 □2 □3 □4 □........

ما السبب الذي دعاك لإجراء/ أو إحالة المريض لإجراء آخر حالة زرع سني ذاتي (منذ ....)؟

□لأنه يعتبر الخيار العلاجي الأفضل للمريض

□لدي تجربة سابقة جيدة، وحصلت على نتائج جيدة

□طريقة للحصول على خبرة جيدة بجراحة الفم

□حالة المريض كانت ملائمة للزرع الذاتي

□لأنها معالجة اقتصادية غير مكلفة للمريض

□غير ذلك..

إذا كانت إجابتك السابقة "لا" لطفاً أجب عن الأسئلة التالية

ما السبب الذي يمنعك من إجراء/ أو إحالة مريض لإجراء وأخذ الزرع السني الذاتي بعين الاعتبار عند وضع خطط المعالجة؟

□وجود بدائل أفضل (الزرعات التيتانية و/أو الجسور)

□السبب المريض: لا يوجد حافز لدى المرضى

□الزرع السني الذاتي ليست معالجة جيدة

□لدي خبرة (و/أو معرفة) قليلة بتقنيات هذه المعالجة

□ليس لدي خبرة (و/أو معرفة) بتقنيات هذه المعالجة

□لدي تجربة سابقة، وحصلت على نتائج سيئة (فشل المعالجة)

□ليس لدي خبرة كافية بالجراحة الفموية

□لأنها معالجة مكلفة وغير مناسبة من ناحية اقتصادية للمريض

□غير ذلك..

من وجهة نظرك، هل تعتبر هذه الطريقة مجدية، وعملية، وفعالة في التعويض عن حالات فقد الأسنان؟ □ نعم □ لا □ لا أعلم

ما التحديات و/أو الاختلاطات التي تخشاها من إجراء هذه المعالجة؟

....
....
....
....
....
